# COPD Exacerbation Biomarkers Validated Using Multiple Reaction Monitoring Mass Spectrometry

**DOI:** 10.1371/journal.pone.0161129

**Published:** 2016-08-15

**Authors:** Janice M. Leung, Virginia Chen, Zsuzsanna Hollander, Darlene Dai, Scott J. Tebbutt, Shawn D. Aaron, Kathy L. Vandemheen, Stephen I. Rennard, J. Mark FitzGerald, Prescott G. Woodruff, Stephen C. Lazarus, John E. Connett, Harvey O. Coxson, Bruce Miller, Christoph Borchers, Bruce M. McManus, Raymond T. Ng, Don D. Sin

**Affiliations:** 1 Centre for Heart Lung Innovation, St. Paul’s Hospital, Vancouver, British Columbia, Canada; 2 Division of Respiratory Medicine, Department of Medicine, St. Paul’s Hospital, Vancouver, British Columbia, Canada; 3 PROOF Center of Excellence, St. Paul’s Hospital, Vancouver, British Columbia, Canada; 4 Ottawa Hospital Research Institute, University of Ottawa, Ottawa, Ontario, Canada; 5 Division of Pulmonary, Critical Care, Sleep and Allergy, University of Nebraska Medical Center, Omaha, Nebraska, United States of America; 6 AstraZeneca, Cambridge, United Kingdom; 7 Division of Respiratory Medicine, Department of Medicine, Vancouver General Hospital and the Institute for Heart and Lung Health, Vancouver, British Columbia, Canada; 8 Division of Pulmonary, Critical Care, Allergy, and Sleep Medicine, Department of Medicine and Cardiovascular Research Institute, University of California San Francisco, San Francisco, California, United States of America; 9 University of Minnesota, Minneapolis, Minnesota, United States of America; 10 GlaxoSmithKline Research and Development, King of Prussia, Pennsylvania, United States of America; 11 University of Victoria-Genome British Columbia Proteomics Centre, Department of Biochemistry and Microbiology, University of Victoria, Victoria, British Columbia, Canada; University of Glasgow, UNITED KINGDOM

## Abstract

**Background:**

Acute exacerbations of chronic obstructive pulmonary disease (AECOPD) result in considerable morbidity and mortality. However, there are no objective biomarkers to diagnose AECOPD.

**Methods:**

We used multiple reaction monitoring mass spectrometry to quantify 129 distinct proteins in plasma samples from patients with COPD. This analytical approach was first performed in a biomarker cohort of patients hospitalized with AECOPD (Cohort A, n = 72). Proteins differentially expressed between AECOPD and convalescent states were chosen using a false discovery rate <0.01 and fold change >1.2. Protein selection and classifier building were performed using an elastic net logistic regression model. The performance of the biomarker panel was then tested in two independent AECOPD cohorts (Cohort B, n = 37, and Cohort C, n = 109) using leave-pair-out cross-validation methods.

**Results:**

Five proteins were identified distinguishing AECOPD and convalescent states in Cohort A. Biomarker scores derived from this model were significantly higher during AECOPD than in the convalescent state in the discovery cohort (p<0.001). The receiver operating characteristic cross-validation area under the curve (CV-AUC) statistic was 0.73 in Cohort A, while in the replication cohorts the CV-AUC was 0.77 for Cohort B and 0.79 for Cohort C.

**Conclusions:**

A panel of five biomarkers shows promise in distinguishing AECOPD from convalescence and may provide the basis for a clinical blood test to diagnose AECOPD. Further validation in larger cohorts is necessary for future clinical translation.

## Introduction

In patients with chronic obstructive pulmonary disease (COPD), fixed airflow limitation often results in symptoms such as dyspnea, cough, and sputum production. The periodic worsening of these symptoms is known as an acute exacerbation (AECOPD), an event that can have lasting effects on lung function [1], respiratory-related quality of life [2], and mortality [3]. Economically, the impact of AECOPD is profound, as annual AECOPD-related costs in the United States alone amount to $30 billion [4]. The diagnosis of an AECOPD, however, largely made on the basis of clinical gestalt, is fraught with uncertainty [5]. Recently, the search for a blood-based biomarker to distinguish AECOPD from clinically stable states has focused on common inflammatory markers such as plasma C-reactive protein (CRP) [6] and serum amyloid protein [7]. Such a restrictive strategy, however, overlooks the heterogeneity of AECOPD in which respiratory viruses, bacterial infection, air pollution, and cardiac dysfunction can all interact through distinct pathways to initiate an event [8–11].

A comprehensive approach to biomarkers could potentially revolutionize the diagnosis and management of AECOPD, ideally revealing biomarkers that could accurately identify AECOPD early in the clinical course to enable intervention. Shotgun proteomics, requiring no *a priori* hypothesis, offers an unbiased platform to detect biomarker candidates and is often used in biomarker discovery, yet the platform is limited by low-throughput, poor accuracy, and suboptimal quantitation during the validation phase of a biomarker study. On the other hand, multiple reaction monitoring-mass spectrometry (MRM-MS) offers an inexpensive, high-throughput platform with the ability to quantify hundreds of proteins based on precursor-product ion pairs [12], and in 2012 was selected by *Nature* as “Method of the Year” [13]. It has since been employed to discover biomarker panels in cystic fibrosis and lung cancer amongst other diseases [14, 15]. To date, MRM-MS has not been applied to COPD and AECOPD, but may provide an opportunity to discover new clinically applicable biomarkers. This study is the first of its kind to employ MRM-MS to identify biomarkers distinguishing AECOPD from periods of clinical stability.

## Methods

### Study Populations

Initial validation of proposed biomarker targets involved72 patients from the previously described and studied cohort evaluating the use of etanercept or prednisone in the treatment of AECOPD (Cohort A, Clinicaltrials.gov NCT00789997) [16]. While 81 subjects were originally included in this study, only 72 had available plasma at both exacerbation and convalescent time points (a confounder analysis revealed no significant demographic differences between patients included and excluded in the final analysis, data not shown). Inclusion criteria were age >35 years, an AECOPD presenting to a physician or emergency department, forced expiratory volume in 1 second(FEV1) ≤70% predicted, FEV1/forced vital capacity (FVC) ≤70%, and ≥10 pack-years smoking history. AECOPD was diagnosed when two of the following three criteria were met: increased dyspnea, sputum volume, and sputum purulence. Patients who had received oral or intravenous corticosteroids in the prior 30 days before entry were excluded, as were patients who had pneumonia, congestive heart failure, or suspected malignancy on the admission chest x-ray. Plasma samples used in this analysis were obtained at baseline and at day 14. The baseline sample was considered to indicate an AECOPD whereas the day 14 sample was used to indicate a convalescent state.

To confirm the biomarkers screened in Cohort A, we used two independent AECOPD cohorts, one a randomized controlled trial with strict criteria for AECOPD definition and the other an observational cohort in which AECOPD were identified by clinicians to better reflect a real world setting. The firstvalidation cohort was a randomized controlled trial evaluating the use of zileuton in the treatment of AECOPD (Cohort B, n = 37, Clinicaltrials.gov NCT00493974) [17]. Briefly, inclusion criteria were age >45 years, admission to the hospital for AECOPD, ≥10 pack-years smoking history, and FEV1<60% predicted. An AECOPD was defined as an acute increase in dyspnea, sputum volume, and/or sputum purulence without an alternative explanation. Subjects with evidence of a lobar pneumonia or pulmonary edema on chest x-ray were excluded. Plasma samples used in this analysis were collected at the beginning of the hospitalization period and at day 30. We considered the initial sample collection at hospitalization to indicate an AECOPD whereas the day 30 samples were used to indicate a convalescent state (as assessed during an outpatient study visit). In both Cohorts A and B, the institutional review boards of each participating site approved the study, as outlined in the original study publications. Written informed consent was provided by each participant in both studies.

The second validation cohort (Cohort C, n = 109, Clinicaltrials.gov NCT02050022) included prospectively enrolled patients admitted to two large teaching hospitals for AECOPD for the purpose of biomarker discovery in AECOPD. Subjects had to be admitted to the hospital with an AECOPD as determined by general internists or pulmonologists, without evidence of pneumonia or heart failure on chest x-ray. Blood samples were collected at the time of admission to the hospital (indicating the AECOPD state) and at either day 30 or 90 following admission (both time points indicating the convalescent state, as assessed during an outpatient study visit). The study was approved by the University of British Columbia Clinical Research Ethics Board (certificate numbers H11-00786 and H13-00790). Written informed consent was provided by each participant in accordance with the Ethics Board.

### Sample Collection

Blood samples from Cohort A were collected in P100 tubes (BD, Franklin Lake, NJ) and stored on ice until processing. Cohort B and Cohort C blood samples were collected in lavender-top EDTA tubes with the plasma layer isolated following centrifugation and stored at -80°C. Blood was processed within two hours of collection and plasma stored at -80°Cuntil the proteomic analysis. Plasma samples were analyzed usingMRM-MS at the UVic Genome BC Proteomics Centre (Victoria, BC, Canada) according to methods described previously [18]. There were 230 peptides measured corresponding to 129 proteins, selected on the basis of both a literature search and from a previous untargeted iTRAQ mass spectrometry analysis on COPD patients. These proteins represented inflammatory cytokines, cell homeostasis, coagulation, lipid metabolism, and immune response. Further details regarding the MRM-MS process, the iTRAQ mass spectrometry analysis, and the peptides measured in this study are provided in S1 File and Table A in S1 File.

### Statistical Analyses

Statistics were performed using R (www.r-project.org) and Bioconductor (www.bioconductor.org). Pre-processing of the MRM-MS data involved several steps. Quantification was done using the ratio of endogenous to heavy labeled standard, and normalization was performed using this ratio for each peptide. To ensure the quality of the data, peptides were excluded from analysis if they had a median relative ratio <0.005, median response <100, or more than two of the standards were outside of the 80–120 range. Peptides with more than 25% missing values (signifying the peptide was below the limit of detection) across all samples were also excluded from analysis. Remaining missing values were imputed with a value equal to half of the minimum peptide level, for each peptide separately. Peptide levels were log 2 transformed, and for proteins with multiple peptides measured, the peptide with the highest levels across most of the samples was chosen to represent the protein.

Proteins were analyzed for differential expression between the patients’ paired AECOPD and convalescent samples, using limma (limma Bioconductor package). A false discovery rate (FDR) <0.01and fold change >1.2 wereused as the criteria for selecting candidate proteins. An elastic net logistic regression model [19](glmnet R package) was applied to the list of candidate proteins to build a classifier or biomarker score. Parameters in this model are chosen to perform stringent feature selection such that the final model excludes features that contain redundant information. The biomarker score was the aggregation of the weighted contributions (linear predictors, denoted here as *w*_*N*_) of each protein in the model to the presence of AECOPD:

$$Biomarker\;score=\;w_{0}+w_{1}*protein_{1}+w_{2}*protein_{2}+\cdots +w_{N}*protein_{N}$$

**Fig 1:**
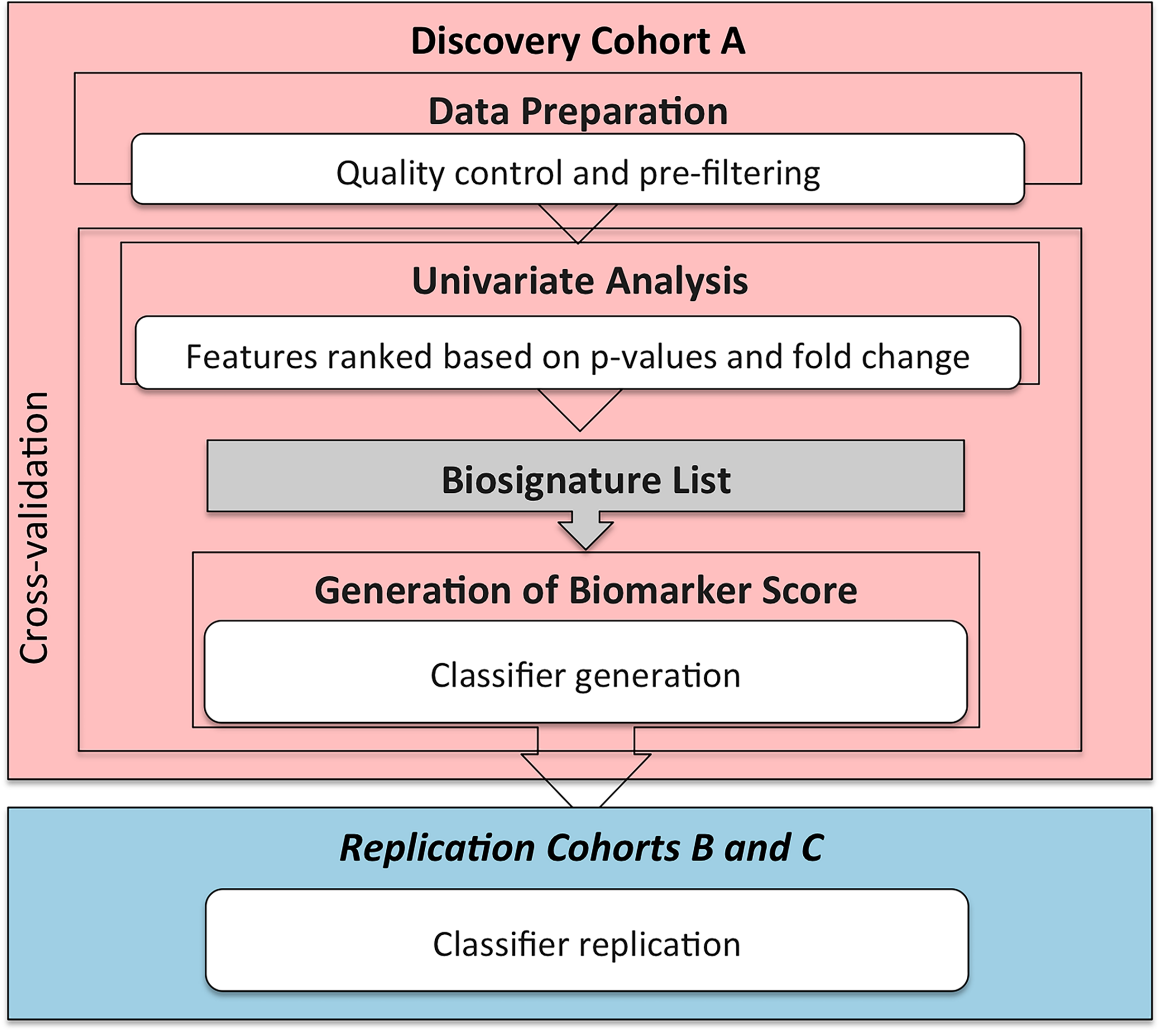
Biomarker Discovery and Replication Strategy. Biomarker discovery steps, applied to Cohort A, are outlined in the pink box. After pre-processing, univariate analysis identifies candidate proteins based on statistically significant differences between AECOPD and convalescent at a false discovery rate <0.01 with a fold change >1.2. An elastic net model is applied to these candidate proteins to generate a final classifier model. This is subsequently followed by replication in Cohorts B and C (blue box).

While the performance characteristics of biomarkers are often evaluated using area under the curve (AUC) statistics from receiver operating characteristics (ROC) curves, this traditional approach tends to overfit the data and gives grossly inflated AUC results that cannot be replicated, particularly in small sample sizes [20]. To circumvent this limitation, we used a leave-pair-out cross-validation (LPOCV) approachto give an unbiased estimate of out-of-sample performance [21, 22]. The cross-validation AUCs (CV-AUC) rather than simple AUCs are thus reported in this study. The LPOCV-based biomarker scores were also used to select decision thresholds, chosen to detect convalescence or exacerbation with at least 90% success, and to optimize Youden’s index given this requirement. The classification model and decision thresholds obtained from Cohort Awere applied to Cohorts B and C for external replication. Fig 1 shows the overall workflow.

## Results

### Cohort Demographics

The demographic characteristics of Cohorts A, B, and C are shown in Table 1. Patients in Cohort C had better lung function than patients in Cohorts A and B, but were also more likely to be current rather than former smokers. Fewer patients in Cohort C were also being treated with inhaled corticosteroids. The majority of patients in all three cohorts were being treated with bronchodilators.

**Table 1:** Demographic Data for Discovery and Validation Cohort.

Characteristic	Cohort A (n = 72)	Cohort B (n = 37)	Cohort C (n = 109)	p-value*
**Age (years)**	67.06 ± 9.28	62.11 ± 8.19	67.79 ± 10.54	0.009
**Male (%)**	37.04	56.76	63.30	0.001
**BMI (kg/m**^**2**^**)**	26.56 ± 7.14	27.04 ± 5.65	27.37 ± 6.88	0.852
**Caucasian (%)**	98.77	59.46	82.41	<0.001
**Smoking Status** **Current (%)** **Former (%)**	23.46 70.37	29.73 70.27	52.29 33.94	<0.001
**Smoking pack-years**	47.85 ± 28.23	47.86 ± 28.02	53.39 ± 36.05	0.476
**FEV1 (L)**^#^	0.94 ± 0.47	1.00 ± 0.62	1.66 ± 0.85	<0.001
**FEV1 (% Predicted)**^#^	34.41 ± 13.87	31.92 ± 15.27	57.19 ± 20.11	<0.001
**FVC (L)**^#^	2.33 ± 1.00	2.35 ± 0.93	2.98 ± 1.16	0.007
**FVC (% Predicted)**^#^	66.65 ± 20.88	56.9 ± 15.7	81.28 ± 19.28	0.001
**FEV1/FVC (%)**^#^	40.78 ± 13.14	41.92 ± 11.61	55.52 ± 13.82	<0.001
**Bronchodilator Use (%)**	100	94.59	95.42	0.134
**Inhaled Corticosteroid Use (%)**	95.00	67.57	44.95	<0.001

Values are reported as mean ± standard deviation or percentages. Abbreviations: BMI—body mass index; FEV1 –forced expiratory volume in 1 second; FVC—forced vital capacity

*P-values were generated using an ANOVA test for continuous variables and chi-square tests for categorical variables.

^#^Spirometry measurements were obtained at the exacerbation time point (upon entry into the study).

### Biomarker Panel Performance

After pre-processing, the MRM-MS data consisted of 55 proteins. Of these, sevenshowed differential levels between exacerbation and convalescent timepoints at a FDR <0.01 with a fold change >1.2 (Table 2).

**Table 2:** Significant Proteins Differentially Expressed in AECOPD Compared to the Convalescent State. Abbreviations: FDR—false discovery rate; AECOPD—acute exacerbations of COPD

Peptide	Protein Name	UniProt ID	Gene Symbol	p-value	FDR	Fold Change	Direction AECOPD Relative to Convalescence
SLAPYAQDTQEK	Apolipoprotein A-IV	P06727	*APOA4*	<0.001	<0.001	1.33	Down
VVEESELAR	Complement component C9	P02748	*C9*	<0.001	<0.001	1.23	Up
SSPVVIDASTAIDAPSNLR	Fibronectin	P02751	*FN1*	<0.001	<0.001	1.23	Down
TAAQNLYEK	Apolipoprotein C-II	P02655	*APOC2*	<0.001	<0.001	1.27	Down
AFVFPK	C-reactive protein	P02741	*CRP*	<0.001	0.001	1.64	Up
GSPAINVAVHVFR	Transthyretin	P02766	*TTR*	<0.001	0.001	1.20	Down
ITLPDFTGDLR	Lipopolysaccharide-binding protein	P18428	*LBP*	0.002	0.005	1.20	Up

**Fig 2:**
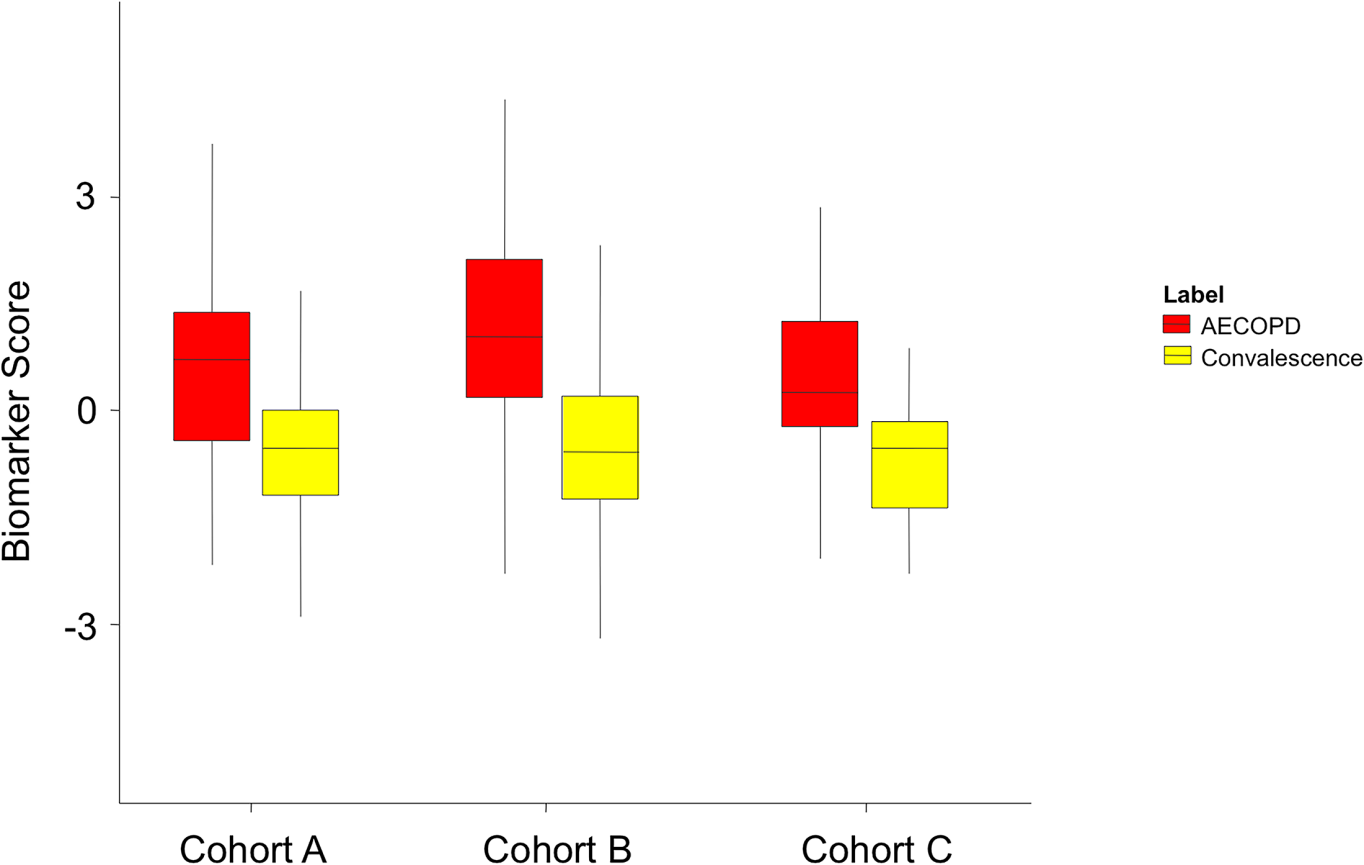
Biomarker Scores Comparing AECOPD to Non-AECOPD States. Biomarker scores for Cohorts A, B, and C are shown as box-and-whisker plots. Biomarker scores were significantly elevated during the time of AECOPD (red) but fell during the convalescent phase (yellow) (Wilcoxon rank sum p-value <0.001 for Cohorts A, B, and C). The convalescent phase scores for Cohorts A, B, and C showed no statistically significant differences.

The final elastic net model consisted of five of these proteins (based on the stringent feature selection of the elastic net regression algorithm in which redundant features are removed, CRP and transthyretin were excluded from the final model). Peptide transitions, collision energies, and fragmentor voltages are provided in Table B in S1 File for these five proteins. Two of the proteins (apolipoprotein C-II and complement component C9) each had two peptides measured and correlation plots between the two peptides for each protein are demonstrated in Fig A in S1 File. A biomarker score based on the weighted contributions of the five proteins to the presence of an AECOPD state was calculated for each cohort. The intercept and specific protein weights contributing to the biomarker score for the 5-protein panel are listed in Table 3. Biomarker scores at each time point for the three cohorts are shown in Fig 2. In each cohort, the biomarker score at exacerbation timepoints was significantly greater than the biomarker score at convalescent time points (Wilcoxon rank sum p-value <0.001 for Cohorts A, B, and C). In addition, the biomarker scores during convalescence in the two replication cohorts were not statistically different from the convalescence biomarker scores in discovery Cohort A. Of note, in Cohort A, the biomarker scores at exacerbation and at convalescence were not statistically significant between the two treatment arms (etanercept vs. prednisolone; p = 0.286 at exacerbation and p = 0.885 at convalescence). The CV-AUCs for the 5-protein panel were 0.73, 0.77, and 0.79 for Cohorts A, B, and C, respectively.

**Table 3:** Biomarker Score Intercept and Specific Protein Weights.

*Biomarker score* = *w*_0_ + w_1_*protein_1_ + *w*_2_*protein_2_ +…+ *w*_N_*protein_*N*_	
Intercept/Protein	*w*
Intercept (*w*_0_)	-0.272
Apolipoprotein A-IV	-1.016
Complement component C9	0.643
Fibronectin	-0.321
Apolipoprotein C-II	-0.225
Lipopolysaccharide-binding protein	0.289

Results from additional subjects and cohorts are available in Fig B in S1 File. For Cohort C, blood samples obtained at day 3 of AECOPD hospitalization demonstrated continued elevation of the biomarker score (no statistically significant difference with the initial AECOPD biomarker score). As well, stable COPD subjects enrolled in Cohort Cwho were not exacerbating hadbiomarker scoresequivalent toconvalescent scores (p = 0.35) (Fig B in S1 File). The panel was further tested in a separate cohort of stable chronic heart failurepatients andhealthy control subjects (see Table C in S1 File for demographics and enrollment criteria)withbiomarker scores from these two groups not significantly different from the convalescent time point.

Positive and negative predictive values in Cohort A for a range of prevalence figures are reported in Table D in S1 File. These are based on a biomarker score cutoff optimized for either a sensitivity ≥90% or a specificity ≥90%.

## Discussion

In this first-ever study employing MRM-MS for biomarker validation in AECOPD, we have generated a promising 5-protein panel for AECOPD diagnosis. The panel appeared to consistently distinguish AECOPD from convalescent states over a range of follow up time periods from 14 days in Cohort A to 90 days in Cohort C. In a “real life” setting (i.e. Cohort C), the biomarker classifier based on these five proteins generated a CV-AUC of 0.79. Once developed, more precise assays that can interrogate these proteins may in future improve CV-AUC values to values >0.8. This will make clinical translation possible [23]. Whether this panel can also identify patients at risk for an imminent AECOPD is yet to be determined. Moreover, how this biomarker panel performs in accordance with proposed patient-reported, symptom-based algorithms for the diagnosis of AECOPD such as the Exacerbations of Chronic Pulmonary Disease Tool (EXACT) [24, 25] is unknown. It should be noted, though, that when tested in non-exacerbating COPD patients who were also enrolled as part of Cohort C and presenting to outpatient follow-up clinics, biomarker scores were no different from AECOPD patients in the convalescent state (see Fig B in S1 File).

Strengths of our study include the use of stringent cross-validation principles to generate CV-AUCs that can better estimate out-of-sample performance, as well as our demonstration of validation in two independent COPD cohorts. The panel performed similarly whether in a randomized controlled trial setting with strict AECOPD criteria or in an observational, “real world” AECOPD cohort. To our knowledge, this approach fills a systematic gap in the COPD biomarker literature in which candidate biomarkers have often been tested in individual cohorts without external replication. Only one recent AECOPD biomarker study, by Bafadhel *et al*., demonstrated external validation of biomarker candidates in an independent cohort [26]. While external replication cohorts can often be difficult to access, the use of cross-validation techniques helps to prevent the overestimation of biomarker performance. Given that data can be overfitted in a statistical model, particularly in small, single cohort studies, the cross-validation approach instead averages performance over multiple iterations of the dataset, providing a more realistic estimate of how a biomarker may perform in a real world setting. There has not yet been an AECOPD biomarker study, however, that has used this approach.

The MRM-MS approach, although previously applied to other disease states [14, 27, 28], marks a departure from traditional methods of biomarker research in AECOPD. Previous attempts at identifying biomarkers have relied on known proteins with available immunoassay platforms, for instance CRP, IL-6, angiopoietin-2, adrenomedullin, and troponin [6, 29–32]. Unfortunately, proteins lacking commercial immunoassays may be overlooked by this strategy. The cost and time required for immunoassay development, however, can be prohibitive [33]. MRM-MS can fill the gap between biomarker discovery and verification by providing a cost-effective platform for quantifying proteins with greater specificity. Moreover, the multiplexing capacity of MRM-MS confers additional advantages.

Using MRM-MS, we identified biological pathways not previously associated with AECOPD pathophysiology. While inflammatory proteins like CRP were indeed differentially expressed in AECOPD, our final biomarker model was not comprised of these proteins, a surprising finding given the attention recently focused on inflammation-related biomarkers. Instead, our panel was notable for the inclusion of two proteins from the cholesterol pathway, apolipoprotein A-IV (APOA4) and apolipoprotein C-II (APOC2) (both decreased in AECOPD). While the associations between AECOPD and cardiovascular comorbidities have long been recognized [8, 34, 35], the specific role of these proteins in the development of AECOPD has not been established. APOA4 is an important constituent of chylomicrons and circulates in plasma either free or bound to high-density lipoproteins (HDL) [36, 37]. While it is primarily associated with lipid metabolism and transport [38, 39], it importantly plays a role in anti-oxidant [40], anti-inflammatory [41, 42] and anti-atherogenic [43, 44] responses. The protein’s relative decrease during AECOPD might suggest that it plays a protective role in the lung as well, although furtherstudies are needed to establish a mechanism. APOC2 circulates in plasma bound to chylomicrons, very low-density lipoproteins (VLDL) and HDL where it serves as an activator of lipoprotein lipase. Deficiencies in APOC2, often inherited as rare autosomal recessive mutations, result in excessive triglyceride levels. Connections between APOC2 to COPD pathogenesis, however, have not yet been established.

There were several limitations to our study. First, the MRM-MS approach is limited by the list of peptides chosen for analysis. It therefore relies on *a priori* assessments and cannot be a truly comprehensive evaluation of all possible biomarkers. Previously, an unbiased proteomics experiment using iTRAQ was performed, which informed the choice of peptides that were interrogated in this study. Nevertheless, given the sensitivity limitations of iTRAQ and other unbiased proteomics platforms, almost certainly there are still undiscovered proteins that probably play a significant role in AECOPD. Secondly, the performance of the protein panel in clinical states that can often be confused with AECOPD, such as congestive heart failureexacerbations, pneumonia, and pulmonary embolism, is unknown but would be critical in determining its performance in patients with non-specific symptoms such as dyspnea. It should be noted, however, that when applying the 5-protein biomarker panel to chronic heart failure patients, the resulting biomarker scores were equivalent to those of convalescent AECOPD patients. This suggests that at least in heart failure, where symptoms can mimic AECOPD, this panel can differentiate between the two conditions. Finally, the influence of treatments such as systemic corticosteroids and antibiotics on the protein signature was not directly tested in this study, but is grounds for future exploration. However, in the discovery cohort, patients who had recently received corticosteroids prior to enrollment were excluded.

In summary, we demonstrate the first application of MRM-MS to biomarker validation in the diagnosis of AECOPD. Not only was this panel able to distinguish AECOPD from the convalescent state in two independent cohorts, but it also revealed potential novel mechanisms for AECOPD by implicating previously unreported cholesterol pathways. For a clinical problem with no current diagnostic test available, our panel may be a significant addition to the management algorithm of COPD.

## Supporting Information

S1 File Table A. Peptides and Corresponding Proteins. Table B. Peptide Transitions, Collision Energies, and Fragmentor Voltages for Biomarker Panel. Table C. Demographic Characteristics of Heart Failure and Healthy Control Patients. Table D. Positive and Negative Predictive Values for Biomarker Panel. Fig A. Correlation Plots Between Peptides for Apolipoprotein C-II and Complement Component C9. Fig B. Biomarker Scores in Chronic Heart Failure Patients and Normal Controls.
(DOCX)

S2 File Dataset forCohort A. (CSV)

S3 File Dataset forCohort B. (CSV)

S4 File Dataset forCohort C. (CSV)
