# Supplementary material for: COPD Exacerbation Biomarkers Validated Using Multiple Reaction Monitoring Mass Spectrometry
Source: PLoS One. 2016 Aug 15;11(8):e0161129. doi: 10.1371/journal.pone.0161129 (PMC4985129; doi:10.1371/journal.pone.0161129)

**COPD Exacerbation Biomarkers Validated Using**

**Multiple Reaction Monitoring Mass Spectrometry**

**Supplemental Materials**

Janice M Leung, Virginia Chen, Zsuzsanna Hollander, Darlene Dai, Scott J Tebbutt, Shawn D Aaron, Kathy L Vandemheen, Stephen I Rennard, J. Mark FitzGerald, Prescott G Woodruff, Stephen C Lazarus, John E Connett, Harvey O Coxson, Bruce Miller, Christoph Borchers, Bruce M McManus, Raymond T Ng, Don D Sin

**Multiple Reaction Monitoring (MRM)-Mass Spectrometry (MS) Methods**

In analytical chemistry, MS is able to identify the chemical composition of a sample by determining the mass-to-charge ratio of analyte ions. Further fragmentation of analyte ions by collision-induced dissociation (tandem MS) allows for protein identification and quantification. Stable isotopes standards (SIS) such as ^13^C, ^15^N, and ^18^O are used as internal standards for the quantification step, in which the relative peak height or peak area of the analyte is compared to the stable isotope-labeled standard. MRM-MS achieves additional specificity, however, by monitoring a precursor ion and one of its collision-induced dissociation-generated product ions while still retaining the precursor and product ions of the stable isotope standard for quantification.

***MRM Assay Development***

Methods for MRM assay development have been previously described (1). First, to identify peptide sequences corresponding to the target protein, a BLAST (Basic Local Assignment Search Tool) search is performed with the goal peptide length between 5 and 25 amino acids. Up to 8 candidate peptides per protein are generated with the list further narrowed based on solubility and liquid chromatography (LC) retention time. SIS versions of the peptides selected are then made. SIS peptides are purified using high-performance LC. The concentration of the synthetic peptide is determined by acid hydrolysis and amino acid analysis. A final SIS mixture is generated by ensuring that the concentration of the SIS peptide is equivalent to the concentration in normal plasma.

***Target Protein Candidates***

230 peptides corresponding to 129 proteins were chosen for this study (see Supplementary Table 1 for the full list). These were chosen based on a literature search and from a previous mass spectrometry analysis on COPD patients enrolled in the Evaluation of COPD Longitudinally to Identify Predict Surrogate Endpoints (ECLIPSE) cohort (GSK Study No. SCO104960, ClinicalTrials.gov NCT00292552) (2).

In the latter analysis, untargeted proteomics with 8-plex isobaric tags for relative and absolute quantification (iTRAQ) was performed on plasma from 300 subjects. iTRAQ analysis was performed in five phases: plasma depletion, trypsin digestion and iTRAQ labeling, high pH reversed phase fractionation, liquid chromatography (LC)-mass spectrometry (MS), and MS data analysis. The 14 most abundant plasma proteins were depleted using a custom-made 5mL avian immunoaffinity column (Genway Biotech, San Diego, CA, USA). Samples were digested with sequencing grade modified trypsin (Promega, Madison, WI, USA) and labeled with iTRAQ reagents 113, 114, 115, 116, 117, 118, 119, and 121 according to the manufacturer’s protocol (Applied Biosystems, Foster City, CA, USA). Each iTRAQ set consisted of seven patient samples and one pool of the patient samples. The reference was randomly assigned to one of the iTRAQ labels. The study samples were randomized to the remaining seven iTRAQ labels by balancing phenotypes between the 43 iTRAQ sets.

High pH reversed phase fractionation was performed with an Agilent 1260 (Agilent, CA, USA) equipped with an XBridge C18 BEH300 (Waters, MA, USA) 250mm X 4.6mm, 5um, 300A HPLC column. The peptide solution was separated by on-line reversed phase liquid chromatography using a Thermo Scientific EASY-nanoLC II system with a reversed-phase pre-column Magic C-18AQ (Michrom BioResources Inc, Auburn, CA) and a reversed-phase nano-analytical column packed with Magic C-18AQ (Michrom BioResources Inc, Auburn, CA), at a flow rate of 300 nl/min. The chromatography system was coupled on-line to an LTQ Orbitrap Velos mass spectrometer equipped with a Nanospray Flex source (Thermo Fisher Scientific, Bremen, Germany). All data was analyzed using ProteinPilot™ Software 3.0 (AB SCIEX, Framingham, MA) and were searched against the Uniprot, version 072010, human database.

A total of 981 proteins were detected in at least one sample. Of these, 84 passed our pre-filtering rule, i.e. to be present in at least 75% of samples. We then compared subjects who had frequent exacerbation (at least 2 exacerbations per year for two years) with those who did not (no exacerbation for two years after blood collection), by means of limma, which identified 43 statistically significant proteins (see Supplemental Table 1).

***MRM-MS Assay***

*Solution and Sample Preparation*

The plasma proteolytic digests were prepared manually as previously described (3). In brief, this involved denaturing, reducing, alkylating, and quenching 10-fold diluted plasma (30 µl) with 1% sodium deoxycholate (30 µL at 10%), 5 mM tris(2-carboxyethyl) phosphine (26.1 µL at 50 mM), 10 mM iodoacetamide (29 µL at 100 mM), and 10 mM dithiothreitol (29 µL at 100 mM; all prepared in 25 mM ammonium bicarbonate), respectively. The protein denaturation and Cys-Cys reduction steps occurred simultaneously for 30 min at 60ºC, while Cys alkylation and iodoacetamide quenching followed sequentially for 30 min at 37ºC. Thereafter, proteolysis was initiated with the addition of TPCK-treated trypsin (10.5 µL at 0.8 mg/mL; Worthington) at a 25:1 substrate:enzyme ratio. After overnight incubation at 37ºC, proteolysis was arrested by the sequential addition of a chilled SIS peptide mixture (30 µL, fmol/µL for the samples) and a chilled FA solution (52.5 µL of 1.9%) to a digest aliquot (117.50 µL). The acid insoluble surfactant was then pelleted by centrifugation and 133.3 µL of each peptide supernatant was removed for solid phase extraction (Oasis HLB µElution Plate 30µm). Following concentration, the eluates were lyophilized to dryness and rehydrated in 50 µL of 0.1% FA (final concentration: 1 µg/µL) for LC-MRM/MS analysis.

*LC-MRM/MS Equipment and Conditions*

Ten µL injections of the plasma digests were separated with a Zorbax Eclipse Plus RP-UHPLC column (2.1 x 150 mm, 1.8 µm particle diameter; Agilent) that was contained within a 1290 Infinity system (Agilent). Peptide separations were achieved at 0.4 mL/min over a 43 min run, via a multi-step LC gradient (1.5-81% mobile phase B; mobile phase compositions: A was 0.1% FA in H_2_O while B was 0.1% FA in ACN). The exact gradient was as follows (time in min, B): 0, 1.5%; 1.5, 6.3%; 16, 13.5%; 18, 13.77%; 33, 22.5%; 38, 40.5%; 39, 81%; 42.9, 81%; 43, 1.5%. The column and autosampler were maintained at 50°C and 4°C, respectively. A post-column equilibration of 4 min was used after each sample analysis. Each individual sample was run in singleton.

The LC system was interfaced to a triple quadrupole mass spectrometer (Agilent 6490) via a standard-flow ESI source, operated in the positive ion mode. The general MRM acquisition parameters employed were as follows: 3.5 kV capillary voltage, 300 V nozzle voltage, 11 L/min sheath gas flow at a temperature of 250°C, 15 L/min drying gas flow at a temperature of 150°C, 30 psi nebulizer gas pressure, 380 V fragmentor voltage, 5 V cell accelerator potential, and unit mass resolution in the quadrupole mass analyzers. Specific LC-MS acquisition parameters were employed for optimal peptide ionization/fragmentation and scheduled MRM. Note that the peptide optimizations were empirically optimized previously by direct infusion of the purified SIS peptides.

*Protein Quantitation*

The MRM data was processed with MassHunter Quantitative Analysis software (Agilent), for verification of peak selection and integration. Reproducibility of the assay was performed according to the CPTAC guidelines for assay validation (https://assays.cancer.gov/about/resources/).

**References**

1. Cohen Freue GV, Borchers CH. Multiple reaction monitoring (MRM): principles and application to coronary artery disease. *Circ-Cardiovasc Gene* 2012; 5: 378.

2. Vestbo J, Anderson W, Coxson HO, Crim C, Dawber F, Edwards L, Hagan G, Knobil K, Lomas DA, MacNee W, Silverman EK, Tal-Singer R. Evaluation of COPD Longitudinally to Identify Predictive Surrogate End-points (ECLIPSE). *Eur Respir J* 2008; 31: 869-873.

3. Percy AJ, Chambers AG, Yang J, Hardie DB, Borchers CH. Advances in multiplexed MRM-based protein biomarker quantitation toward clinical utility. *Biochim Biophys Acta* 2014; 1844: 917-926.

**S1 Table. Peptides and Corresponding Proteins**

*Denotes peptides discovered from a previous untargeted iTRAQ mass spectrometry analysis performed on the ECLIPSE cohort.

| **Peptide** | **Protein Name** | **Accession Number** | **Gene Symbol** |
| --- | --- | --- | --- |
| IDAVYEAPQEEK | 72 kDa type IV collagenase | P08253 | MMP2 |
| IIGYTPDLDPETVDDAFAR | 72 kDa type IV collagenase | P08253 | MMP2 |
| DSYVGDEAQSK | Actin, alpha cardiac muscle 1 | P68032 | ACTC |
| SYELPDGQVITIGNER | Actin, alpha cardiac muscle 1 | P68032 | ACTC |
| TSLGSDSSTQAK | Adenylate cyclase type 9 | O60503 | ADCY9 |
| GDIGETGVPGAEGPR | Adiponectin | Q15848 | ADIPO |
| IFYNQQNHYDGSTGK | Adiponectin | Q15848 | ADIPO |
| IAPQLSTEELVSLGEK | Afamin | P43652 | AFAM |
| LPNNVLQEK | Afamin | P43652 | AFAM |
| AVLDVFEEGTEASAATAVK | Alpha-1-antichymotrypsin | P01011 | AACT |
| NLAVSQVVHK | Alpha-1-antichymotrypsin | P01011 | AACT |
| ITPNLAEFAFSLYR | Alpha-1-antitrypsin | P01009 | A1AT |
| LSITGTYDLK | Alpha-1-antitrypsin | P01009 | A1AT |
| ATWSGAVLAGR | Alpha-1B-glycoprotein | P04217 | A1BG |
| LETPDFQLFK | Alpha-1B-glycoprotein | P04217 | A1BG |
| DFLQSLK* | Alpha-2-antiplasmin | P08697 | A2AP |
| LGNQEPGGQTALK* | Alpha-2-antiplasmin | P08697 | A2AP |
| APHGPGLIYR* | Alpha-2-HS-glycoprotein | P02765 | FETUA |
| HTLNQIDEVK* | Alpha-2-HS-glycoprotein | P02765 | FETUA |
| LLIYAVLPTGDVIGDSAK | Alpha-2-macroglobulin | P01023 | A2MG |
| TEHPFTVEEFVLPK | Alpha-2-macroglobulin | P01023 | A2MG |
| ALQDQLVLVAAK* | Angiotensinogen | P01019 | ANGT |
| DDLYVSDAFHK* | Antithrombin-III | P01008 | ANT3 |
| FATTFYQHLADSK* | Antithrombin-III | P01008 | ANT3 |
| ATEHLSTLSEK | Apolipoprotein A-I | P02647 | APOA1 |
| SPELQAEAK | Apolipoprotein A-II | P02652 | APOA2 |
| SLAPYAQDTQEK | Apolipoprotein A-IV | P06727 | APOA4 |
| FPEVDVLTK | Apolipoprotein B-100 | P04114 | APOB |
| ILGEELGFASLHDLQLLGK | Apolipoprotein B-100 | P04114 | APOB |
| TAAQNLYEK* | Apolipoprotein C-II | P02655 | APOC2 |
| TYLPAVDEK* | Apolipoprotein C-II | P02655 | APOC2 |
| FSEFWDLDPEVR | Apolipoprotein C-III | P02656 | APOC3 |
| GWVTDGFSSLK | Apolipoprotein C-III | P02656 | APOC3 |
| VTEPISAESGEQVER | Apolipoprotein L1 | O14791 | APOL1 |
| WWTQAQAHDLVIK | Apolipoprotein L1 | O14791 | APOL1 |
| FVTVQTISGTGALR | Aspartate aminotransferase, mitochondrial | P00505 | AATM |
| ATVVYQGER | Beta-2-glycoprotein 1 | P02749 | APOH |
| PDNGFVNYPAKPTLYYK | Beta-2-glycoprotein 1 | P02749 | APOH |
| IQVYSR | Beta-2-microglobulin | P61769 | B2MG |
| VNHVTLSQPK | Beta-2-microglobulin | P61769 | B2MG |
| ALEQDLPVNIK* | Beta-Ala-His dipeptidase | Q96KN2 | CNDP1 |
| SVVLIPLGAVDDGEHSQNEK* | Beta-Ala-His dipeptidase | Q96KN2 | CNDP1 |
| AFVFPK | C-reactive protein | P02741 | CRP |
| ESDTSYVSLK | C-reactive protein | P02741 | CRP |
| EDVYVVGTVLR* | C4b-binding protein alpha chain | P04003 | C4BPA |
| LSLEIEQLELQR* | C4b-binding protein alpha chain | P04003 | C4BPA |
| YEIVVEAR | Cadherin-5 | P33151 | CADH5 |
| YTFVVPEDTR | Cadherin-5 | P33151 | CADH5 |
| ESISVSSEQLAQFR | Carbonic anhydrase 1 | P00915 | CAH1 |
| VLDALQAIK | Carbonic anhydrase 1 | P00915 | CAH1 |
| EALIQFLEQVHQGIK* | Carboxypeptidase N catalytic chain | P15169 | CBPN |
| IVQLIQDTR* | Carboxypeptidase N catalytic chain | P15169 | CBPN |
| LVGGLHR | CD5 antigen-like | O43866 | CD5L |
| GAYPLSIEPIGVR | Ceruloplasmin | P00450 | CERU |
| IYHSHIDAPK | Ceruloplasmin | P00450 | CERU |
| ELDESLQVAER* | Clusterin | P10909 | CLUS |
| EPQDTYHYLPFSLPHR* | Clusterin | P10909 | CLUS |
| LANLTQGEDQYYLR* | Clusterin | P10909 | CLUS |
| SALVLQYLR* | Coagulation factor IX | P00740 | FA9 |
| VSVSQTSK* | Coagulation factor IX | P00740 | FA9 |
| AEVDDVIQVR | Coagulation factor V | P12259 | FA5 |
| VAQVIIPSTYVPGTTNHDIALLR | Coagulation factor VII | P08709 | FA7 |
| VSQYIEWLQK | Coagulation factor VII | P08709 | FA7 |
| NLFLTNLDNLHENNTHNQEK | Coagulation factor VIII | P00451 | FA8 |
| ETYDFDIAVLR* | Coagulation factor X | P00742 | FA10 |
| TGIVSGFGR* | Coagulation factor X | P00742 | FA10 |
| LHEAFSPVSYQHDLALLR | Coagulation factor XII | P00748 | FA12 |
| VVGGLVALR | Coagulation factor XII | P00748 | FA12 |
| AVPPNNSNAAEDDLPTVELQGVVPR | Coagulation factor XIII A chain | P00488 | F13A |
| STVLTIPEIIIK | Coagulation factor XIII A chain | P00488 | F13A |
| IQTHSTTYR | Coagulation factor XIII B chain | P05160 | F13B |
| LIENGYFHPVK | Coagulation factor XIII B chain | P05160 | F13B |
| PAFSAIR* | Complement C1q subcomponent subunit A | P02745 | C1QA |
| SLGFCDTTNK* | Complement C1q subcomponent subunit A | P02745 | C1QA |
| FQSVFTVTR* | Complement C1q subcomponent subunit C | P02747 | C1QC |
| TNQVNSGGVLLR* | Complement C1q subcomponent subunit C | P02747 | C1QC |
| GLTLHLK* | Complement C1r subcomponent | P00736 | C1R |
| GYGFYTK* | Complement C1r subcomponent | P00736 | C1R |
| SYPPDLR* | Complement C1r subcomponent | P00736 | C1R |
| TLDEFTIIQNLQPQYQFR* | Complement C1r subcomponent | P00736 | C1R |
| VSVHPDYR* | Complement C1r subcomponent | P00736 | C1R |
| SDFSNEER* | Complement C1s subcomponent | P09871 | C1S |
| TNFDNDIALVR* | Complement C1s subcomponent | P09871 | C1S |
| DHENELLNK | Complement C2 | P06681 | CO2 |
| HAFILQDTK | Complement C2 | P06681 | CO2 |
| TGLQEVEVK | Complement C3 | P01024 | CO3 |
| DHAVDLIQK | Complement C4-A | P0C0L4 | CO4A |
| VGDTLNLNLR | Complement C4-A | P0C0L4 | CO4A |
| VLSLAQEQVGGSPEK | Complement C4-A | P0C0L4 | CO4A |
| GFVVAGPSR | Complement component C6 | P13671 | CO6 |
| ELSHLPSLYDYSAYR | Complement component C7 | P10643 | CO7 |
| LIDQYGTHYLQSGSLGGEYR | Complement component C7 | P10643 | CO7 |
| SYTSHTNEIHK | Complement component C7 | P10643 | CO7 |
| SLPVSDSVLSGFEQR | Complement component C8 gamma chain | P07360 | CO8G |
| VQEAHLTEDQIFYFPK | Complement component C8 gamma chain | P07360 | CO8G |
| LSPIYNLVPVK | Complement component C9 | P02748 | CO9 |
| VVEESELAR | Complement component C9 | P02748 | CO9 |
| EELLPAQDIK | Complement factor B | P00751 | CFAB |
| THHDGAITER | Complement factor D | P00746 | CFAD |
| SSNLIILEEHLK* | Complement factor H | P08603 | CFAH |
| SSQESYAHGTK* | Complement factor H | P08603 | CFAH |
| HGNTDSEGIVEVK* | Complement factor I | P05156 | CFAI |
| IVIEYVDR* | Complement factor I | P05156 | CFAI |
| AQLLQGLGFNLTER | Corticosteroid-binding globulin | P08185 | CBG |
| HLVALSPK | Corticosteroid-binding globulin | P08185 | CBG |
| TLDEILQEK | Cyclin-dependent kinase 11A | Q9UQ88 | CD11A |
| TSNLLLSHAGILK | Cyclin-dependent kinase 11A | Q9UQ88 | CD11A |
| ALDFAVGEYNK | Cystatin-C | P01034 | CYTC |
| ALQVVR | Cystatin-C | P01034 | CYTC |
| ELPSLQHPNEQK | Extracellular matrix protein 1 | Q16610 | ECM1 |
| NVALVSGDTENAK | Extracellular matrix protein 1 | Q16610 | ECM1 |
| LVVLPFPK* | Fetuin-B | Q9UGM5 | FETUB |
| VNDAQEYR* | Fetuin-B | Q9UGM5 | FETUB |
| AHYGGFTVQNEANK | Fibrinogen beta chain | P02675 | FIBB |
| QGFGNVATNTDGK | Fibrinogen beta chain | P02675 | FIBB |
| YEASILTHDSSIR | Fibrinogen gamma chain | P02679 | FIBG |
| HTSVQTTSSGSGPFTDVR* | Fibronectin | P02751 | FINC |
| SSPVVIDASTAIDAPSNLR* | Fibronectin | P02751 | FINC |
| GYHLNEEGTR | Fibulin-1 | P23142 | FBLN1 |
| SQETGDLDVGGLQETDK | Fibulin-1 | P23142 | FBLN1 |
| TGYYFDGISR | Fibulin-1 | P23142 | FBLN1 |
| TGAQELLR* | Gelsolin | P06396 | GELS |
| PGGGFVPNFQLFEK* | Glutathione peroxidase 3 | P22352 | GPX3 |
| VGYVSGWGR | Haptoglobin | P00738 | HPT |
| TYFPHFDLSHGSAQVK | Hemoglobin subunit alpha | P69905 | HBA |
| VGAHAGEYGAEALER | Hemoglobin subunit alpha | P69905 | HBA |
| LYLVQGTQVYVFLTK* | Hemopexin | P02790 | HEMO |
| NFPSPVDAAFR* | Hemopexin | P02790 | HEMO |
| GETHEQVHSILHFK* | Heparin cofactor 2 | P05546 | HEP2 |
| NYNLVESLK* | Heparin cofactor 2 | P05546 | HEP2 |
| SVNDLYIQK* | Heparin cofactor 2 | P05546 | HEP2 |
| SPLNDFQVLR | Hepatocyte growth factor-like protein | P26927 | HGFL |
| DGYLFQLLR* | Histidine-rich glycoprotein | P04196 | HRG |
| FLNVLSPR | Insulin-like growth factor-binding protein 3 | P17936 | IBP3 |
| YGQPLPGYTTK | Insulin-like growth factor-binding protein 3 | P17936 | IBP3 |
| NLIAAVAPGAFLGLK* | Insulin-like growth factor-binding protein complex acid labile subunit | P35858 | ALS |
| VAGLLEDTFPGLLGLR* | Insulin-like growth factor-binding protein complex acid labile subunit | P35858 | ALS |
| ETAVDGELVVLYDVK* | Inter-alpha-trypsin inhibitor heavy chain H2 | P19823 | ITIH2 |
| FLHVPDTFEGHFDGVPVISK* | Inter-alpha-trypsin inhibitor heavy chain H2 | P19823 | ITIH2 |
| SPEQQETVLDGNLIIR | Inter-alpha-trypsin inhibitor heavy chain H4 | Q14624 | ITIH4 |
| ISTLSCENK | Interleukin-18 | Q14116 | IL18 |
| IITGLLEFEVYLEYLQNR | Interleukin-6 | P05231 | IL6 |
| VGSALFLSHNLK* | Kallistatin | P29622 | KAIN |
| DIPTNSPELEETLTHTITK | Kininogen-1 | P01042 | KNG1 |
| TVGSDTFYSFK | Kininogen-1 | P01042 | KNG1 |
| DLLHVLAFSK | Leptin | P41159 | LEP |
| YSENSTTVIR | Leptin receptor | P48357 | LEPR |
| GLQYAAQEGLLALQSELLR | Lipopolysaccharide-binding protein | P18428 | LBP |
| ITLPDFTGDLR | Lipopolysaccharide-binding protein | P18428 | LBP |
| LGSFEGLVNLTFIHLQHNR* | Lumican | P51884 | LUM |
| LPSGLPVSLLTLYLDNNK* | Lumican | P51884 | LUM |
| SLEDLQLTHNK* | Lumican | P51884 | LUM |
| SLEYLDLSFNQIAR* | Lumican | P51884 | LUM |
| FNSVPLTDTGHER | Macrophage colony-stimulating factor 1 | P09603 | CSF1 |
| APGELEHGLITFSTR | Mannan-binding lectin serine protease 1 | P48740 | MASP1 |
| TEGQFVDLTGNR | Mannose-binding protein C | P11226 | MBL2 |
| LGLGADVAQVTGALR | Matrix metalloproteinase-9 | P14780 | MMP9 |
| SLGPALLLLQK | Matrix metalloproteinase-9 | P14780 | MMP9 |
| FVGTPEVNQTTLYQR | Metalloproteinase inhibitor 1 | P01033 | TIMP1 |
| GFQALGDAADIR | Metalloproteinase inhibitor 1 | P01033 | TIMP1 |
| ELTLEDLK | Monocyte differentiation antigen CD14 | P08571 | CD14 |
| FPAIQNLALR | Monocyte differentiation antigen CD14 | P08571 | CD14 |
| STLSVGVSGTLVLLQGAR | Monocyte differentiation antigen CD14 | P08571 | CD14 |
| IANVFTNAFR | Myeloperoxidase | P05164 | PERM |
| VVLEGGIDPILR | Myeloperoxidase | P05164 | PERM |
| AAPAPAPPPEPERPK | Myosin light chain 3 | P08590 | MYL3 |
| PSLSHLLSQYYGAGVAR* | N-acetylmuramoyl-L-alanine amidase | Q96PD5 | PGRP2 |
| TDCPGDALFDLLR* | N-acetylmuramoyl-L-alanine amidase | Q96PD5 | PGRP2 |
| ALPAVETQAPTSLATK | Peptidase inhibitor 16 | Q6UXB8 | PI16 |
| ATAVVDGAFK | Peroxiredoxin-2 | P32119 | PRDX2 |
| GLFIIDGK | Peroxiredoxin-2 | P32119 | PRDX2 |
| SSGLVSNAPGVQIR | Phosphatidylcholine-sterol acyltransferase | P04180 | LCAT |
| AVEPQLQEEER | Phospholipid transfer protein | P55058 | PLTP |
| FLEQELETITIPDLR | Phospholipid transfer protein | P55058 | PLTP |
| TSLEDFYLDEER* | Pigment epithelium-derived factor | P36955 | PEDF |
| TVQAVLTVPK* | Pigment epithelium-derived factor | P36955 | PEDF |
| VSEGNHDIALIK | Plasma kallikrein | P03952 | KLKB1 |
| LLDSLPSDTR | Plasma protease C1 inhibitor | P05155 | IC1 |
| AVVEVDESGTR | Plasma serine protease inhibitor | P05154 | IPSP |
| FSIEGSYQLEK | Plasma serine protease inhibitor | P05154 | IPSP |
| VILGAHQEVNLEPHVQEIEVSR* | Plasminogen | P00747 | PLMN |
| DEISTTDAIFVQR* | Plasminogen activator inhibitor 1 | P05121 | PAI1 |
| FSLETEVDLR* | Plasminogen activator inhibitor 1 | P05121 | PAI1 |
| AFIQLWAFDAVK* | Protein AMBP | P02760 | AMBP |
| ETLLQDFR* | Protein AMBP | P02760 | AMBP |
| HHGPTITAK* | Protein AMBP | P02760 | AMBP |
| ETSNFGFSLLR | Protein Z-dependent protease inhibitor | Q9UK55 | ZPI |
| LFDEINPETK | Protein Z-dependent protease inhibitor | Q9UK55 | ZPI |
| DQYYNIDVPSR | Proteoglycan 4 | Q92954 | PRG4 |
| GFGGLTGQIVAALSTAK | Proteoglycan 4 | Q92954 | PRG4 |
| ELLESYIDGR* | Prothrombin | P00734 | THRB |
| ETAASLLQAGYK* | Prothrombin | P00734 | THRB |
| YWGVASFLQK* | Retinol-binding protein 4 | P02753 | RET4 |
| AEFAEVSK | Serum albumin | P02768 | ALBU |
| LVNEVTEFAK | Serum albumin | P02768 | ALBU |
| FRPDGLPK | Serum amyloid A-4 protein | P35542 | SAA4 |
| GPGGVWAAK | Serum amyloid A-4 protein | P35542 | SAA4 |
| AYSLFSYNTQGR | Serum amyloid P-component | P02743 | SAMP |
| IQNILTEEPK* | Serum paraoxonase/arylesterase 1 | P27169 | PON1 |
| SFNPNSPGK* | Serum paraoxonase/arylesterase 1 | P27169 | PON1 |
| IALGGLLFPASNLR | Sex hormone-binding globulin | P04278 | SHBG |
| VVLSQGSK | Sex hormone-binding globulin | P04278 | SHBG |
| GGTLGTPQTGSENDALYEYLR* | Tetranectin | P05452 | TETN |
| AVLHIGEK* | Thyroxine-binding globulin | P05543 | THBG |
| FSISATYDLGATLLK* | Thyroxine-binding globulin | P05543 | THBG |
| TLYETEVFSTDFSNISAAK* | Thyroxine-binding globulin | P05543 | THBG |
| IPVVLPEDEGIYTAFASNIK | Titin | Q8WZ42 | TITIN |
| VAGESAEPEPEPEADYYAK | Transforming growth factor beta-1 | P01137 | TGFB1 |
| VEQHVELYQK | Transforming growth factor beta-1 | P01137 | TGFB1 |
| AADDTWEPFASGK* | Transthyretin | P02766 | TTHY |
| GSPAINVAVHVFR* | Transthyretin | P02766 | TTHY |
| LHIDEMDSVPTVR | Vascular cell adhesion protein 1 | P19320 | VCAM1 |
| LAGLGLQQLDEGLFSR | Vasorin | Q6EMK4 | VASN |
| SLTLGIEPVSPTSLR | Vasorin | Q6EMK4 | VASN |
| YLQGSSVQLR | Vasorin | Q6EMK4 | VASN |
| ELPEHTVK* | Vitamin D-binding protein | P02774 | VTDB |
| THLPEVFLSK* | Vitamin D-binding protein | P02774 | VTDB |
| LGEYDLR | Vitamin K-dependent protein C | P04070 | PROC |
| TFVLNFIK | Vitamin K-dependent protein C | P04070 | PROC |
| YLDWIHGHIR | Vitamin K-dependent protein C | P04070 | PROC |
| SFQTGLFTAAR* | Vitamin K-dependent protein S | P07225 | PROS |
| VYFAGFPR* | Vitamin K-dependent protein S | P07225 | PROS |
| DFAEHLLIPR | Vitamin K-dependent protein Z | P22891 | PROZ |
| ENFVLTTAK | Vitamin K-dependent protein Z | P22891 | PROZ |
| DVWGIEGPIDAAFTR* | Vitronectin | P04004 | VTNC |
| FEDGVLDPDYPR* | Vitronectin | P04004 | VTNC |
| IGWPNAPILIQDFETLPR | von Willebrand factor | P04275 | VWF |
| ILAGPAGDSNVVK | von Willebrand factor | P04275 | VWF |
| AGEVQEPELR* | Zinc-alpha-2-glycoprotein | P25311 | ZA2G |
| YSLTYIYTGLSK* | Zinc-alpha-2-glycoprotein | P25311 | ZA2G |

**S2 Table. Peptide Transitions, Collision Energies, and Fragmentor Voltages for Biomarker Panel**

| **Compound Name** | **Precursor Ion** | **Product Ion** | **Fragmentor** | **Collision Energy** |  |  |
| --- | --- | --- | --- | --- | --- | --- |
| ITLPDFTGDLR.heavy | 629.3398 | 522.274 | 380 | 13 | Qualifier | y9++ |
| ITLPDFTGDLR.heavy | 629.3398 | 465.7319 | 380 | 17 | Qualifier | y8++ |
| ITLPDFTGDLR.heavy | 629.3398 | 930.456 | 380 | 13 | Quantifier | y8 |
| ITLPDFTGDLR.light | 624.3398 | 517.274 | 380 | 13 | Qualifier | y9++ |
| ITLPDFTGDLR.light | 624.3398 | 460.7319 | 380 | 17 | Qualifier | y8++ |
| ITLPDFTGDLR.light | 624.3398 | 920.456 | 380 | 13 | Quantifier | y8 |
| SLAPYAQDTQEK.heavy | 679.8381 | 579.78 | 380 | 17 | Qualifier | y10++ |
| SLAPYAQDTQEK.heavy | 679.8381 | 272.161 | 380 | 17 | Qualifier | b3 |
| SLAPYAQDTQEK.heavy | 679.8381 | 544.2615 | 380 | 17 | Quantifier | y9++ |
| SLAPYAQDTQEK.light | 675.8381 | 575.78 | 380 | 17 | Qualifier | y10++ |
| SLAPYAQDTQEK.light | 675.8381 | 272.161 | 380 | 17 | Qualifier | b3 |
| SLAPYAQDTQEK.light | 675.8381 | 540.2615 | 380 | 17 | Quantifier | y9++ |
| SSPVVIDASTAIDAPSNLR.heavy | 641.674 | 782.4036 | 380 | 17 | Qualifier | y7 |
| SSPVVIDASTAIDAPSNLR.heavy | 641.674 | 448.2478 | 380 | 9 | Qualifier | y8++ |
| SSPVVIDASTAIDAPSNLR.heavy | 641.674 | 596.3396 | 380 | 17 | Quantifier | y5 |
| SSPVVIDASTAIDAPSNLR.light | 638.3406667 | 772.4036 | 380 | 17 | Qualifier | y7 |
| SSPVVIDASTAIDAPSNLR.light | 638.3406667 | 443.2478 | 380 | 9 | Qualifier | y8++ |
| SSPVVIDASTAIDAPSNLR.light | 638.3406667 | 586.3396 | 380 | 17 | Quantifier | y5 |
| TAAQNLYEK.heavy | 523.28 | 447.24 | 380 | 17 | Qualifier | y3 |
| TAAQNLYEK.heavy | 523.28 | 437.24 | 380 | 13 | Qualifier | y7++ |
| TAAQNLYEK.heavy | 523.28 | 873.46 | 380 | 17 | Quantifier | y7 |
| TAAQNLYEK.light | 519.28 | 439.24 | 380 | 17 | Qualifier | y3 |
| TAAQNLYEK.light | 519.28 | 433.24 | 380 | 13 | Qualifier | y7++ |
| TAAQNLYEK.light | 519.28 | 865.46 | 380 | 17 | Quantifier | y7 |
| VVEESELAR.heavy | 521.2767 | 585.3236 | 380 | 13 | Qualifier | y5 |
| VVEESELAR.heavy | 521.2767 | 328.1872 | 380 | 5 | Qualifier | b3 |
| VVEESELAR.heavy | 521.2767 | 843.4088 | 380 | 13 | Quantifier | y7 |
| VVEESELAR.light | 516.2767 | 575.3236 | 380 | 13 | Qualifier | y5 |
| VVEESELAR.light | 516.2767 | 328.1872 | 380 | 5 | Qualifier | b3 |
| VVEESELAR.light | 516.2767 | 833.4088 | 380 | 13 | Quantifier | y7 |

**S3 Table. Demographic Characteristics of Heart Failure and Healthy Control Patients**

| **Characteristic** | **Heart Failure Cohort* (n=218)** | **Healthy Control**  **(n=49)** |
| --- | --- | --- |
| Age (mean ± standard deviation) | 64.9 ± 11.0 | 45.9 ± 14.4 |
| Male (%) | 75.7 | 24.5 |
| White Race (%) | 76.9 | 79.5 |
| Smoking Status  Never (%)  Current (%)  Former (%) | 37.2  11.0  51.8 | N/A |

*****Entry criteria into the Heart Failure Cohort were 1) age 19 years or older; 2) either heart failure with preserved ejection fraction (≥50%) or heart failure with ejection fraction ≤40%; and 3) New York Heart Association Class II, III, or IV. Exclusion criteria included: 1) a recent cardiac even (<6 weeks from an acute coronary syndrome, heart failure exacerbation, or other cardiac-related hospitalization); 2) severe mitral or aortic stenosis; 3) hereditary cardiomyopathy or congenital heart abnormality; and 4) cardiac amyloidosis or sarcoidosis.

**S4 Table. Positive and Negative Predictive Values for Biomarker Panel**

| **Optimization** | **Sensitivity** | **Specificity** | **Biomarker Score Cutoff** | **Prevalence** | **NPV** | **PPV** |
| --- | --- | --- | --- | --- | --- | --- |
| Sensitivity ≥90% | 0.90 | 0.37 | -0.753 | 0.01 | 99.7% | 1.4% |
|  |  |  |  | 0.02 | 99.5% | 2.8% |
|  |  |  |  | 0.03 | 99.2% | 4.2% |
|  |  |  |  | 0.04 | 98.9% | 5.6% |
|  |  |  |  | 0.05 | 98.6% | 7.0% |
|  |  |  |  | 0.10 | 97.1% | 13.7% |
|  |  |  |  | 0.15 | 95.5% | 20.2% |
|  |  |  |  | 0.20 | 93.8% | 26.4% |
|  |  |  |  | 0.25 | 91.9% | 32.3% |
| Specificity ≥90% | 0.47 | 0.91 | 1.231 | 0.01 | 99.4% | 4.9% |
|  |  |  |  | 0.02 | 98.8% | 9.3% |
|  |  |  |  | 0.03 | 98.2% | 13.5% |
|  |  |  |  | 0.04 | 97.6% | 17.4% |
|  |  |  |  | 0.05 | 97.0% | 21.0% |
|  |  |  |  | 0.10 | 93.9% | 35.9% |
|  |  |  |  | 0.15 | 90.6% | 47.1% |
|  |  |  |  | 0.20 | 87.2% | 55.8% |
|  |  |  |  | 0.25 | 83.6% | 62.7% |

Abbreviations: NPV: Negative predictive value; PPV: Positive predictive value

**S1 Fig. Correlation Plots Between Peptides for Apolipoprotein C-II and Complement Component C9.**

Two proteins in the biomarker panel (apolipoprotein C-II and complement component C9) had two peptides measured. The correlation plots for peptide levels are shown for A) apolipoprotein C-II and B) complement component C9. Both demonstrate high correlation between the peptides.

**S2 Fig. Biomarker Scores in Chronic Heart Failure Patients and Normal Controls.**

Biomarker scores for the 5-protein panel are shown for a cohort of chronic heart failure patients (n=218) and for healthy controls (n=49) to compare with the three AECOPD cohorts. Scores for the chronic heart failure patients and for the healthy controls were not statistically different from the convalescent scores of the discovery Cohort A (p=0.07 and p=0.13, respectively). Included in this figure are also Day 3 and non-exacerbating COPD control patients in Cohort C, demonstrating that biomarker scores remain high in the immediate AECOPD period and that non-exacerbating COPD controls have similar biomarker scores to convalescent patients.

**S1 Fig.**

**A.**


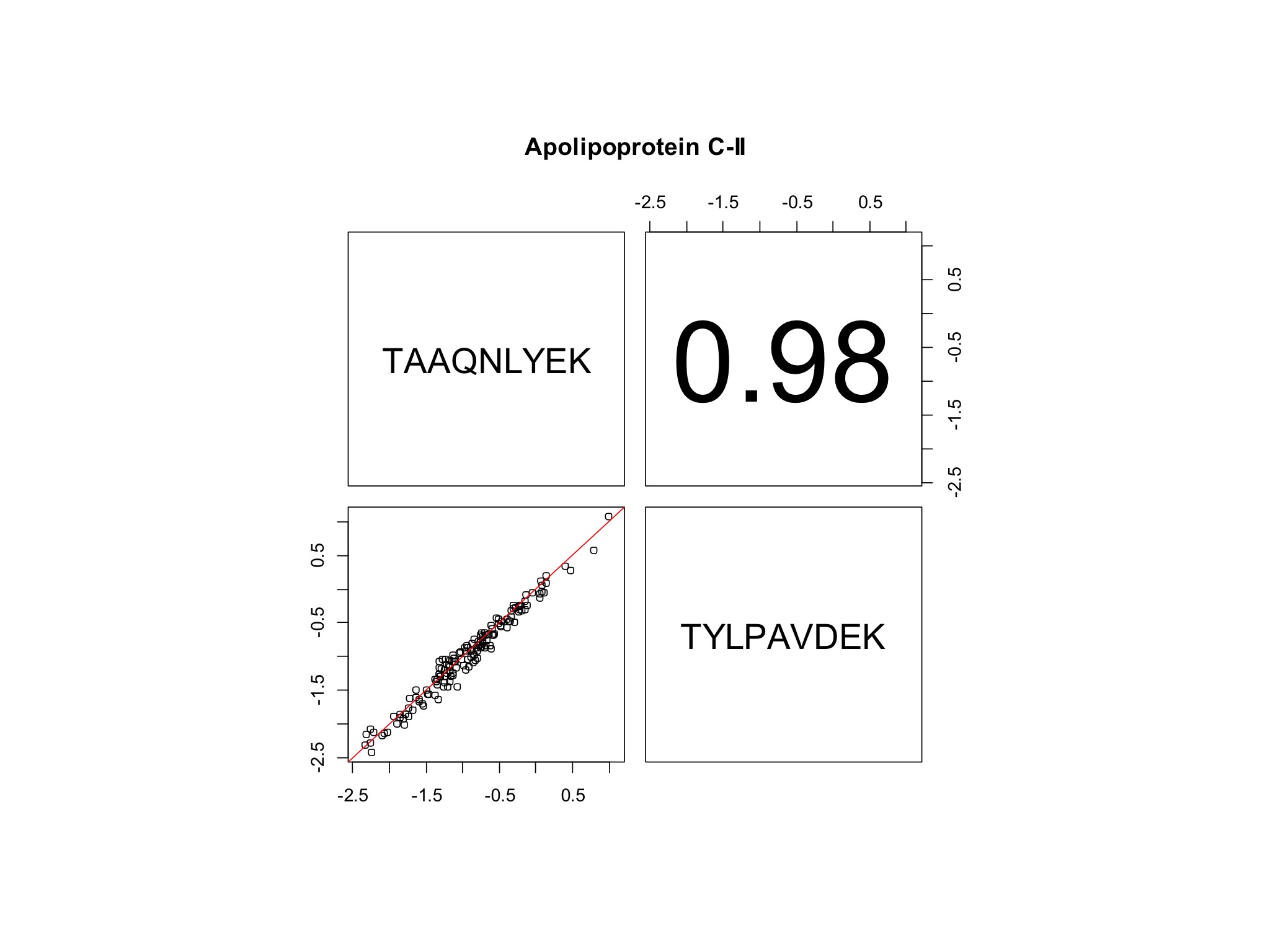


**B.**


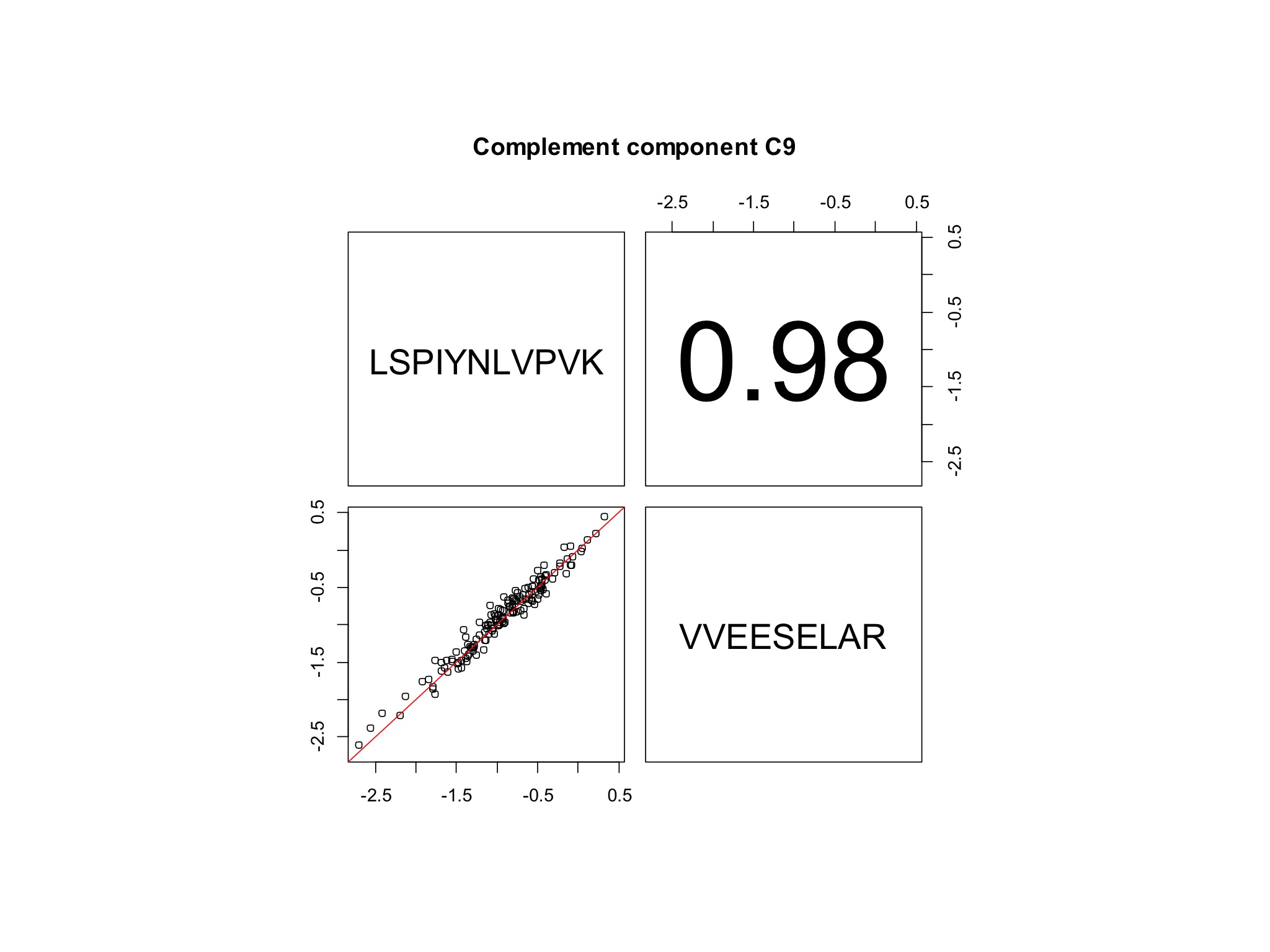


**S2 Fig.**


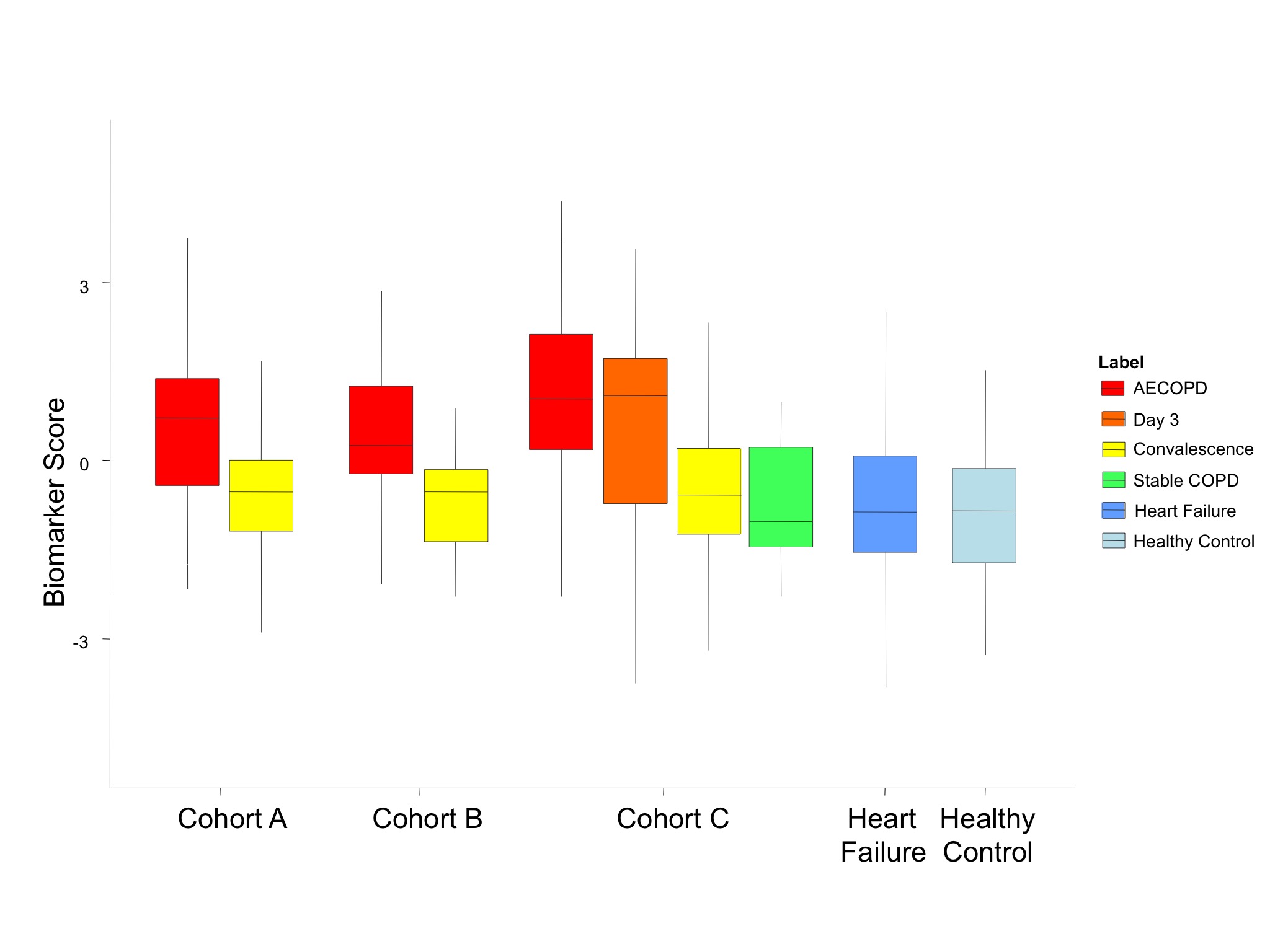

Supplement: S1 File — Table A. Peptides and Corresponding Proteins. Table B. Peptide Transitions, Collision Energies, and Fragmentor Voltages for Biomarker Panel. Table C. Demographic Characteristics of Heart Failure and Healthy Control Patients. Table D. Positive and Negative Predictive Values for Biomarker Panel. Fig A. Correlation Plots Between Peptides for Apolipoprotein C-II and Complement Component C9. Fig B. Biomarker Scores in Chronic Heart Failure Patients and Normal Controls. (DOCX) [file pone.0161129.s001.docx]
